# Micro-CT Assessment of Internal and External Void Formation in Class II Restorations of Primary Molars Using Bulk-Fill Composites

**DOI:** 10.3390/ma18112621

**Published:** 2025-06-03

**Authors:** Ralitsa Gigova, Krasimir Hristov

**Affiliations:** Department of Pediatric Dentistry, Faculty of Dental Medicine, Medical University of Sofia, 1431 Sofia, Bulgaria; r.bogovska@fdm.mu-sofia.bg

**Keywords:** bulk-fill materials, void formation, micro-CT, primary molars, class II restoration

## Abstract

This study aimed to assess the formation of internal and external voids in class II restorations of primary molars using bulk-fill composites with different viscosities through micro-CT analysis. Standardized class II cavities were prepared on 50 extracted intact primary molars. The teeth were restored with bulk-fill materials of varying viscosity: SDR, Tetric EvoCeram bulk-fill, Viscalor bulk, Cention forte, and a control group (Dyract XP). They were then scanned using a computed microtomograph. The volumes of the internal and external voids were quantified and expressed as percentages (%) of the total restoration volume. The data were analyzed using one-way ANOVA, followed by Tukey’s test (α = 0.05). The detected external and internal voids ranged from 0.19% to 0.62%. The data indicated no significant difference in the formation of external voids among the various bulk-fill materials or the control group (*p* > 0.05). Significantly fewer internal voids were observed with more flowable materials and when heat was applied (*p* < 0.05). The highest percentage of internal and external voids was observed when the layering restorative technique was used. It was concluded that in class II bulk-fill composite restorations in primary dentition, the percentages of both external and internal voids were relatively small compared with the entire volume of the restoration and decreased when more flowable materials were used.

## 1. Introduction

Resin composites are commonly used in restorative dentistry because of their favorable adhesive, mechanical, and thermal properties [1]. Direct composite restorations must endure occlusal loading and masticatory forces, maintain stability in a biological environment, be biocompatible, and prevent the formation of microcracks, cracks, and voids [2]. Additionally, they should be easy to use [3]. The history of dental composites began in the 1960s with the introduction of resin-based materials, combining polymeric matrices with inorganic fillers [4]. Over the decades, advancements in filler technology, including the incorporation of micro- and nano-sized particles, have enhanced their mechanical, thermal, and tribological properties [4]. Today, nano-filled composites, such as those incorporating hydroxyapatite, offer superior performance [4].

Conventional resin composites require placement in layers, with thicknesses not exceeding 2 mm [5]. This limitation is crucial for ensuring thorough polymerization of the monomer particles and achieving a strong adhesive bond [6]. However, this layering technique can lead to the formation of voids within the material or at the tooth surface as well as prolong the clinical time needed to complete the restoration [7].

To address the challenges associated with the layering technique, bulk-fill composites have emerged in the dental market over the past decade [8]. These materials can be applied in thicknesses up to three times greater than those of conventional composites [9]. Bulk-fill composites must fulfill two primary requirements: achieving an adequate degree of monomer conversion throughout the thickness of the material and possessing the capacity to compensate for or dissipate polymerization stress within the cavity boundaries [10].

Cention Forte is an alkasite material used for posterior restorations. It is designed to release fluoride, calcium, and hydroxide ions, which can contribute to the remineralization of tooth structures and provide an antibacterial effect [11]. It demonstrates superior mechanical properties and a significantly better sealing ability than other bulk-fill materials, as evidenced by lower silver nitrate dye penetration, indicating fewer voids and better marginal integrity [11,12].

SDR is a flowable bulk-fill composite resin commonly used in dental restorations. It has been shown to exhibit pronounced energy dissipation and damping characteristics, making it a promising material for biomimetic restoration of viscoelastic dentin structures in primary teeth [13]. It has been demonstrated that this material exhibits excellent adaptation to cavity walls and generally lower porosity compared with other bulk-fill composites, which is crucial for the longevity and integrity of restorations [14].

Tetric EvoCeram bulk fill is a high-viscosity, nano-hybrid composite resin suitable for restorations in primary molars [15]. Its ability to be placed in 4-mm increments without compromising polymerization makes it particularly advantageous for pediatric dentistry, where minimizing chair time is crucial [15]. Tetric EvoCeram bulk fill demonstrates good adaptation to cavity walls with minimal void formation. Micro-computed tomography and scanning electron microscopy studies have shown that it has lower porosity compared with some other bulk-fill composites [14,16].

Viscalor bulk fill is a thermoviscous bulk-fill composite resin that employs thermoviscous technology, which improves its mechanical behavior and marginal integrity. It has been demonstrated to have high marginal integrity in dentin and exhibit low internal void formation compared with other bulk-fill composites. Additionally, Viscalor bulk fill has favorable physico-mechanical properties such as low volumetric polymerization shrinkage and polymerization stress, contributing to its overall good performance in dental restorations [17,18].

Compomers combine the properties of both glass ionomers and composite resins. This material is not universally considered the gold standard for restorations in primary dentition. However, it has been recognized as a suitable and effective material for primary teeth in multiple clinical studies because it releases fluoride ions, which can help in caries prevention, and has good handling characteristics, making it suitable for pediatric patients [19,20]. The formation of voids in Dyract compomer restorations is considered minimal. Studies have shown that the material performs well regarding marginal adaptation and integrity, with no significant marginal gaps observed in SEM evaluations [21].

The ability to restore an entire cavity with a single layer of material offers numerous advantages [9]. Primarily, this approach is less time-consuming and reduces the risk of technical errors, such as inclusion of air voids within the material or contamination between layers. It also facilitates better control over humidity during the procedure [9]. This offers considerable benefits in restoration in primary dentition. Primary teeth exhibit unique histological and structural characteristics, such as thinner enamel and dentin, which can affect the performance of dental materials in pediatric dentistry. Comprehending these distinctions is crucial for enhancing treatment protocols for primary teeth [22,23].

In composite dental restorations, voids refer to small air pockets or gaps within or around the restorative material. These voids can compromise the restoration’s integrity, strength, and longevity, potentially leading to clinical issues like secondary caries, fracture, or failure [24]. Voids are categorized into internal voids and external voids based on their locations within the restoration. Internal voids are air bubbles or gaps trapped within the bulk of the composite material during placement or curing, while external voids are gaps or defects located at the interface between the composite restoration and the tooth structure (enamel or dentin) or on the outer surface of the restoration [24].

Bulk-fill materials can be categorized based on their viscosity into two types: low-flow and high-flow composites [25]. The latest generation of bulk-fill composites features an increased filler content, which enhances their mechanical properties [25]. High-viscosity bulk-fill composites require longer curing times compared with low-viscosity composites in order to achieve optimal mechanical characteristics [26]. Low-viscosity materials enhance the wetting effect on the cavity walls, which improves marginal adaptation, reduces the number of voids, and minimizes microleakage [27,28]. The viscosity of the material is significantly influenced by factors such as the resin composition, filler particle fraction, filler size distribution, silane pretreatment, and temperature [29].

Applying high-viscosity composite materials can be challenging, often leading to poor adaptation to cavity walls or the formation of voids [18]. To mitigate this risk, the use of flowable materials, along with techniques to enhance their flowability—such as the application of heat—can be beneficial [18].

Different types of analysis and equipment can be used to assess the internal and external voids in bulk-fill restorations. Scanning electron microscopy is employed to analyze the external marginal seal and adaptation of the restoration. It provides detailed images of the surface morphology. It can be used with energy-dispersive X-ray spectroscopy to assess the chemical composition at the tooth–restoration interface [18,30].

Optical coherence tomography is used for real-time imaging of gap formation during the polymerization process. It allows for the visualization of voids and gaps within the restoration and can be used to compare different restorative techniques [31].

Our study uses micro-computed tomography (μCT) to evaluate internal voids and marginal adaptation in bulk-fill restorations, as μCT can provide high-resolution, three-dimensional images that allow for precise quantification of voids and gaps within the restoration and at the tooth–restoration interface [30].

Voids, whether internal or external, undermine the mechanical integrity of restorations, increasing the likelihood of clinical complications such as fractures, microleakage, and secondary caries [24]. These risks are amplified in class II cavities in primary teeth due to their complex geometry and the distinct properties of primary dentin, including elevated moisture content and higher tubule density, which can exacerbate void formation during material placement.

This study aims to evaluate, through micro-CT analysis, the formation of internal and external voids in class II restorations of primary molars using bulk-fill materials with different viscosities.

## 2. Materials and Methods

### 2.1. Sample Preparation

The study included 50 primary molars extracted immediately prior to their physiological exfoliation. Informed consent was signed from the parents of the children whose teeth were used in the study. On each molar, one clinician prepared two standardized class II cavities, specifically mesio-occlusal and disto-occlusal, measuring 3 mm × 4 mm × 2 mm for the bucco-oral, occluso-gingival, and mesio-distal dimensions, respectively. The cavity preparations were performed with a high-speed handpiece under water cooling conditions, using a diamond cylindrical bur with a flat tip, rounded edges, and an abrasiveness of 125 µm (Strauss Diamond, Palm Coast, FL, USA). The cavities were finished with fine diamond burs of the same shape but with an abrasiveness of 45 µm (Strauss Diamond, Palm Coast, FL, USA). New burs were used for the preparation of each cavity. The buccal and oral walls of the cavities were designed to be parallel to each other and perpendicular to the gingival base, while the axial wall was also perpendicular to the gingival base. The internal edges were rounded.

The samples were restored using bulk-fill materials of varying viscosities, with a compomer serving as the control group. Dyract XP was selected as the control for its common use in pediatric dentistry, offering fluoride release and a simplified application suitable for primary teeth. This contrasts with conventional composites, which are typically used in layered techniques for permanent teeth, ensuring relevance to our study’s pediatric context.

Details on the materials used, including their manufacturers, compositions, viscosities, and applied restorative techniques, are presented in Table 1.

### 2.2. Restorative Technique

**Figure 1:**
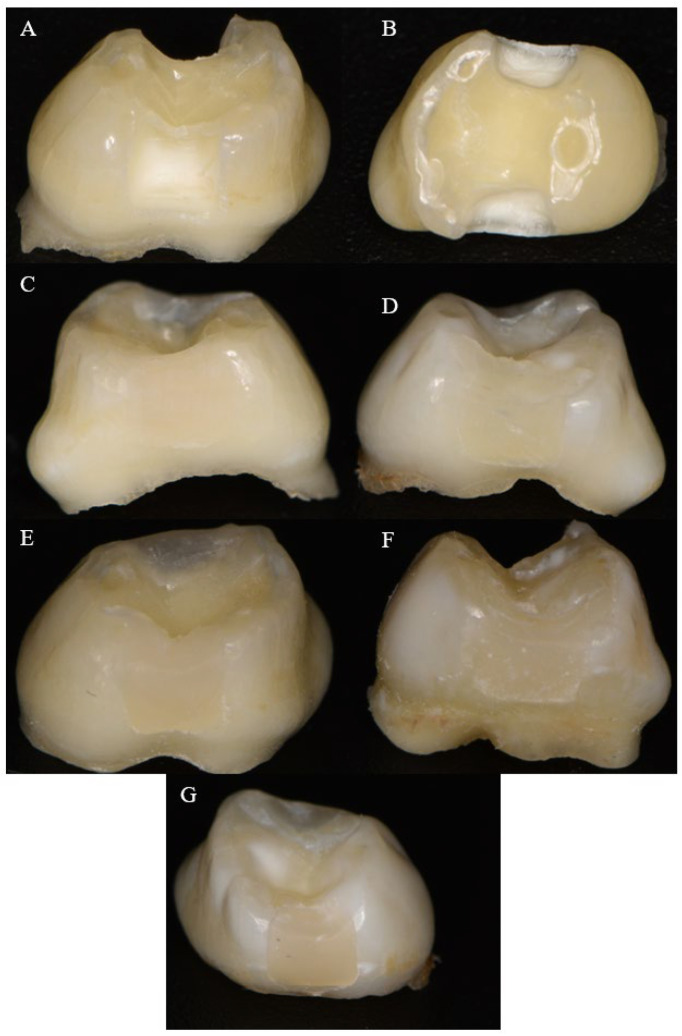
Proximal (**A**) and occlusal (**B**) views of the prepared cavities and final restorations with SDR (**C**), Tetric EvoCeram bulk-fill (**D**), Viscalor bulk (**E**), Cention Forte (**F**), and Duract XP (**G**).

Transparent celluloid matrices (SuperMat Celluloid Matrices, Kerr, Kloten, Switzerland) were adapted to fit around each tooth. The samples were then randomly divided into five groups, with each containing 10 teeth. The teeth from four of the groups were etched with 37% orthophosphoric acid (3M™ Scotchbond™ Universal Adhesive, 3M ESPE, Athlone, Ireland) for 30 s. After etching, the gel was rinsed off, and the cavities were dried. A layer of adhesive (Adhese Uni System Viva Pen, Ivoclar Vivadent, Schaan, Liechtenstein) was applied using a microapplicator, followed by polymerization with a light-curing lamp (Freelight 2 Elipar™, 3MESPE, Athlone, Ireland) at a light intensity of 1.470 mV/sm^2^ for 20 s. As all materials, except the alkasite material, utilized camphorquinone as the photoinitiator, photopolymerization was consistently performed under standardized conditions: 20 s of exposure at a light intensity of 1470 mV/cm^2^ and a wavelength range of 430–480 nm. The teeth from the groups were restored using SDR, Tetric EvoCeram bulk fill, Viscalor bulk, or Dyract XP. The cavities from the last group were coated with Cention Primer (Ivoclar Vivadent, Schaan, Liechtenstein), following the manufacturer’s instructions and restored with Cention Forte. The restorations were then finished and polished using discs with decreasing levels of abrasiveness (Sof-Lex Pop-on, 3M ESPE, St. Paul, MN, USA). Figure 1 presents images of the standardized cavity and the completed restorations with different bulk-fill materials.

### 2.3. Scanning Procedure

The teeth were analyzed using a SkyScan 1272 computed X-ray microtomograph (Bruker, Kontich, Belgium). The scanning parameters were set to a voltage of 100 kV, current of 80 μA, a 1.0-mm copper filter, a pixel size of 9 μm, a rotation step of 0.45°, a full sample rotation of 360°, and an exposure time of 1000 ms for each projection. The average scanning time for each sample was approximately 20 min. The NRecon software (version 2.2.0.6, Bruker, Billerica, MA, USA) converted the tooth projections into cross-sectional slices using the following settings: ring artifacts set to 4.0, smoothing set to 0, beam hardening at 45%, and proper compensation for misalignment.

### 2.4. Void Analysis

**Figure 2:**
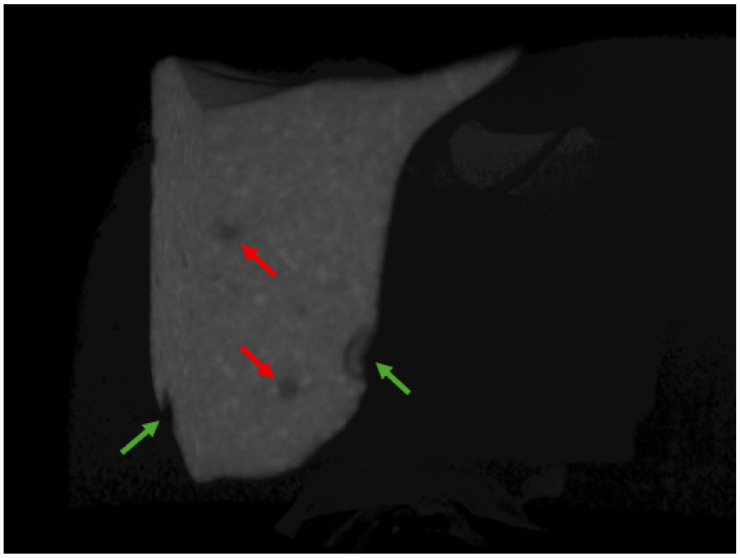
An image of a clipped restoration, showing internal voids (marked with red arrows) and external voids (marked with green arrows).

The two restorations, mesial and distal, were assessed separately. The analysis was conducted after reconstructing the specimens using CTAn visualization software (Bruker, Kontich, Belgium), version 1.23.01. This involved examining the 3D microarchitecture of each filling. Internal voids were defined as air pockets or gaps fully enclosed within the bulk of the composite restoration, whereas external voids were defined as gaps located at the interface between the restoration and the tooth structure (enamel or dentin) or on the external surface of the restoration (Figure 2). Void segmentation was performed using the previously mentioned software, with a gray color lower threshold ranging between 35 and 75 for the different materials and an upper threshold of 255 to distinguish voids (low-density air spaces) from the higher-density restorative material and tooth structure. The volumes of both the internal and external voids were calculated as a percentage (%) of the total volume of the restoration.

### 2.5. Sample Size Calculation, Statistical Methods, and Data Processing

The samples were divided into five groups, composed of 10 teeth per group (n = 10), which was based on the specialized literature [18,32]. The Shapiro–Wilk test was utilized to assess the normality of the data. One-way ANOVA was performed to evaluate the impact of different types of bulk-fill resin composites on void formation. Tukey’s test was employed for pairwise comparisons. The significance level was set at 5% (*p* = 0.05). Statistical analysis was conducted with SPSS v.19.0 statistics computer software (SPSS Inc., Chicago, IL, USA).

## 3. Results

**Figure 3:**
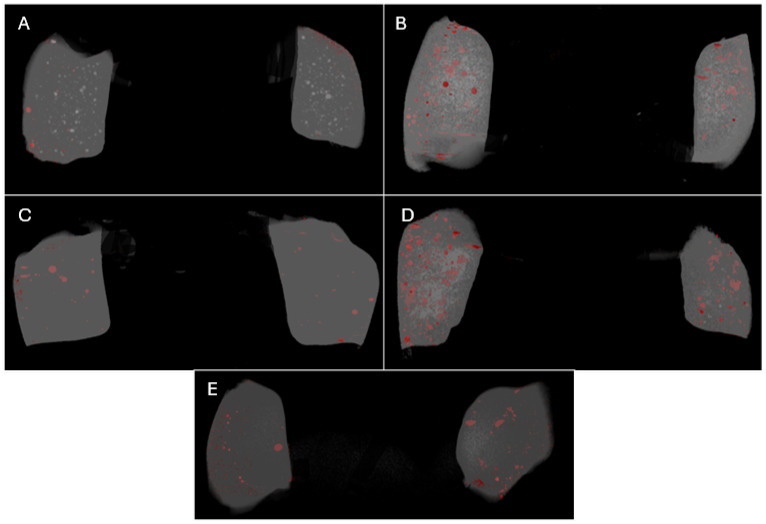
Representative three-dimensional Micro-CT images of external and internal voids in restorative materials with different viscosities: SDR (**A**), Tetric EvoCeram bulk-fill (**B**), Viscalor bulk (**C**), Cention Forte (**D**), and Duract XP (**E**).

Three-dimensional reconstruction of the scanned samples is presented in Figure 3.

**Figure 4:**
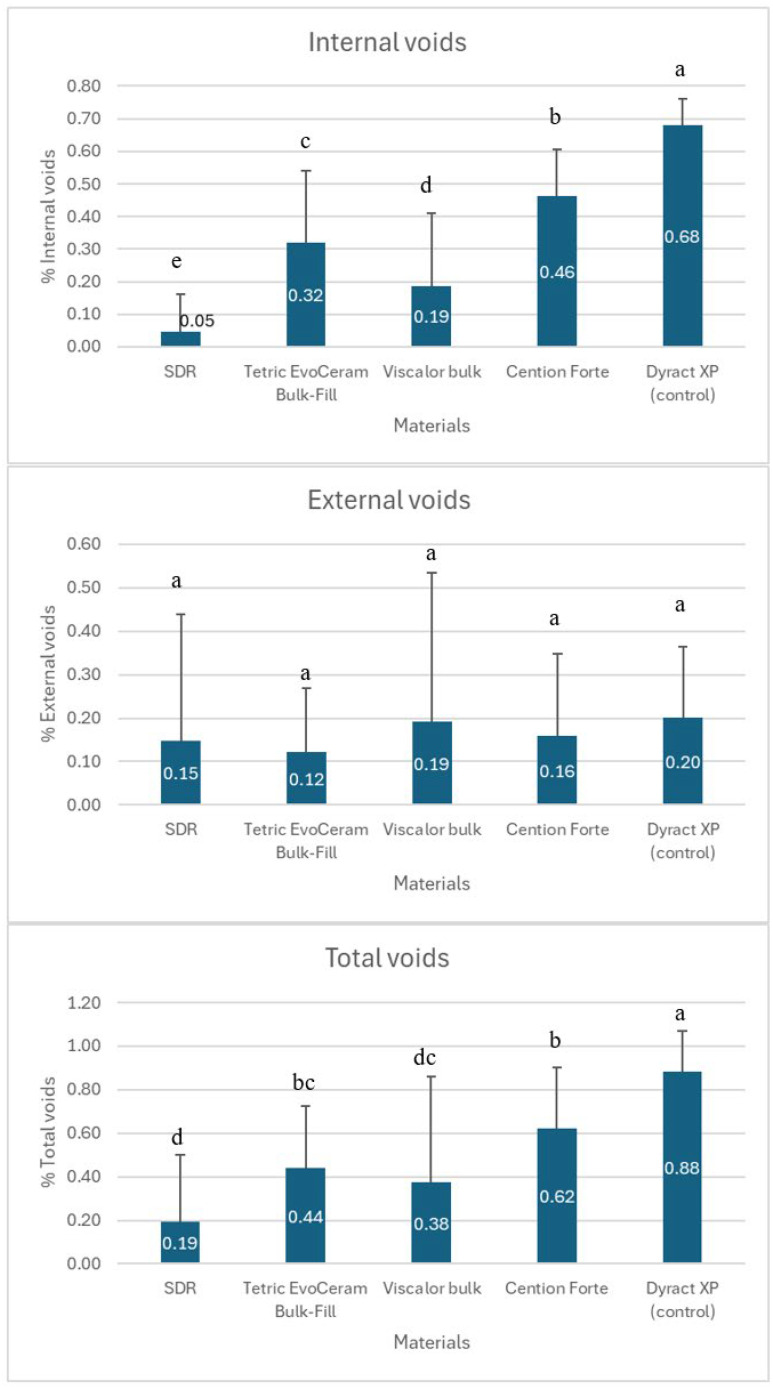
Internal, external, and total voids as a percentage of the total filling volume in bulk-fill materials with different viscosities. Different lowercase letters (a–e) indicate significant differences.

Figure 4 presents the estimates of external and internal voids in the studied materials.

All tested restorative materials exhibited varying percentages of void formation. Most defects were noted in the control group that employed a layered restoration technique. The data indicate that there were no statistically significant differences among the various groups regarding external void formation (*p* > 0.05). However, differences did exist among all tested materials concerning the percentage of internal void formation (*p* < 0.05). The highest percentage of internal voids was observed when the layering restorative technique was used with Dyract XP. The Cention and Tetric groups, the two materials with moderate and high viscosities, displayed high percentages of internal voids, while Viscalor exhibited intermediate results for void formation. The group of teeth restored with SDR showed the lowest levels of both external and internal voids due to the lowest viscosity of the material.

## 4. Discussion

This study aimed to quantify the percentage of internal and external voids in class II restorations using bulk-fill composites of varying viscosities in primary teeth and to evaluate the impact of material viscosity and application techniques on void formation. The results indicated that a higher number of voids formed with a high-viscosity material and a layering technique.

The presence of internal and external voids in dental bulk-fill composites poses a significant issue due to their adverse effects on the mechanical properties and durability of the restorations [18]. Internal voids in bulk-fill composites can occur due to several factors, including the material’s viscosity, the method of application, and the polymerization process. A study by Sá et al. demonstrated that preheating bulk-fill composites significantly reduced the percentage of internal voids compared with composites applied at room temperature [18]. This study utilized a thermoviscous bulk-fill material preheated to 68 °C. The data (Figure 4) showed that the Viscalor group had significantly fewer internal voids compared with the Tetric and Cention groups. However, while the differences were minor, Viscalor did not outperform SDR with respect to the volume of internal voids formed. Some researchers suggest that the injection technique is more effective in minimizing internal defects compared with the conventional incremental restoration technique [18].

This suggests that preheating enhances material adaptation, a critical factor in pediatric dentistry, where efficient application is essential due to limited patient cooperation. The AAPD guidelines emphasize the importance of durable restorations in primary teeth to withstand masticatory forces until exfoliation [33]. Our findings support the use of preheated thermoviscous materials to minimize internal voids, which can weaken mechanical properties and increase fracture risk.

In the present study, the layering restorative technique with the compomer material Dyract XP was used as a control group. The results showed that the percentage of internal defects in the other groups was significantly lower than that in the control group.

External voids at the restoration interface are primarily influenced by how well the composite adheres to and adapts to the cavity walls. Sampaio et al. showed that the extent of volumetric polymerization shrinkage and the formation of voids depend on the specific material used. Generally, bulk-fill materials exhibit better adaptation and fewer voids compared with conventional composites [31]. However, the relationship between polymerization shrinkage and void formation has not been established for all materials [31]. In the present study, no bulk-fill material was significantly better in terms of external voids, and there were no statistically significant differences among the groups (Figure 3 and Figure 4). The lack of significant differences in external voids may be attributed to the standardized cavity preparation and application protocols used in this study, which minimized variability in adhesion. However, external voids remain a concern in pediatric dentistry, as they can lead to microleakage and secondary caries, compromising restoration longevity [22]. The AAPD recommends materials and techniques that reduce microleakage to ensure long-term success in primary tooth restorations [33]. Our findings suggest that while bulk-fill composites perform adequately, further optimization of application techniques is needed to align with these clinical standards.

The method used to apply the material significantly influences the formation of defects in restorations. Some researchers have found that the monobloc technique with sound activation leads to a higher internal porosity compared with other methods, indicating that sonic vibration can increase both the number and volume of closed pores [16]. In contrast, Demirel et al. discovered that the use of preheating combined with sonic application methods can reduce void formation, with preheating generally resulting in fewer voids [34]. In the current study, the total percentage of voids present in the thermoviscous material was significantly lower than that observed in alkasite, as well as those in high-viscosity bulk-fill materials (Figure 4).

Internal voids can weaken the mechanical properties of composites, leading to reduced strength and an increased risk of material fracture. Over time, these voids may serve as stress concentration zones, which could facilitate crack formation and eventual failure of the restoration. Moreover, internal voids can disrupt the polymerization process, resulting in incomplete curing and decreased hardness, ultimately diminishing the durability of the composite [35].

On the other hand, external voids on the surface of a dental restoration can cause microleakage, permitting bacteria and oral fluids to infiltrate these areas. This can result in secondary caries, postoperative sensitivity, and, ultimately, treatment failure. External voids may contribute to staining and compromise the aesthetic outcome of the restoration [24,36].

This study revealed that the percentage of pores in bulk-fill composites was remarkably low, ranging from 0.19% to 0.62% among the groups studied. In contrast, the group restored with the layering technique exhibited 0.88% voids. This indicates that bulk-fill composites are a viable alternative to traditional filling materials for children and supports the hypothesis that bulk-fill materials reduce internal defects compared with conventional incremental techniques, consistent with clinical preferences for efficient restorative procedures in pediatric patients [33]. However, it is important to note certain limitations. The study focused on exfoliated primary molars, which may not accurately represent the clinical condition, particularly in the context of pediatric patient treatment.

Limitations: Our study, which employed an ex vivo design using extracted exfoliating molars, has several limitations that may have impacted our findings.

First, the ex vivo model does not replicate the dynamic oral environment. Unlike in vivo conditions, the experiments lacked saliva flow, pH fluctuations, microbial activity, and masticatory forces. These factors can significantly affect material adhesion and the formation of restoration voids, limiting the clinical applicability of our results.

Second, exfoliating molars from pediatric patients differ structurally and compositionally from functional primary teeth. These molars often have thinner enamel, less mineralized dentin, or altered surface properties due to the exfoliation process, which may influence adhesion and void formation. Consequently, our findings may be most relevant to specific pediatric contexts and less applicable to other tooth types or developmental stages.

Third, the ex vivo design does not account for tooth aging processes, such as enamel wear, dentin sclerosis, or changes in organic content. These factors can affect material adhesion and restoration longevity, reducing the generalizability of our results to older populations or long-term clinical outcomes.

Finally, the controlled laboratory conditions did not simulate the mechanical and thermal stresses of the oral environment, such as cyclic loading from chewing or temperature changes from foods and beverages. These untested variables may also influence material performance and void formation.

## 5. Conclusions

All tested bulk-fill restorative materials exhibited some formation of both internal and external voids, with the SDR composite material demonstrating the lowest percentage of internal voids. However, no bulk-fill material significantly outperformed the others in minimizing external void formation. Given the overall low void percentages observed, bulk-fill composites represent a promising alternative to conventional restorative materials and techniques in pediatric dentistry.

Key Points:

- The SDR bulk-fill composite exhibited the fewest internal voids, suggesting superior adaptation within the restoration bulk in primary molars.
- External void formation was comparable across all tested bulk-fill materials, indicating that no single material offers a significant advantage in interfacial adaptation.
- The low overall void percentages (expressed as the percentage of the total restoration volume) support the clinical viability of bulk-fill composites for class II restorations in pediatric dentistry, offering efficiency and reduced technique sensitivity.

## Figures and Tables

**Table 1:** Materials and their compositions, viscosities, and restorative techniques used in the study.

Groups	Manufacturer	Restorative Technique	Viscosity	Composition
Group 1 SDR	Dentsply Sirona, Konstanz, Germany	The material was injected directly into the cavity from the compule at room temperature. The tip of the compule was placed in contact with the gingival base, and the material was extruded using the bulk-fill technique.	Low-viscosity bulk-fill resin composite	Modified UDMA, TEGDMA, dimethacrylate and trimethacrylate resin, silanated bariumalumino-fluoroborosilicate glass, silanated strontium alumino-fluoro-silicate glass, surface treated fume silicas, ytterbium fluoride, synthetic inorganic iron oxide pigments, and titanium dioxide
Group 2 Tetric EvoCeram bulk-fill	Ivoclar Vivadent, Schaan, Liechtenstein	The material was applied in one portion with the bulk-fill technique and condensed and shaped using modeling instruments (OptraSculpt & OptraSculpt Pad System Kit, Ivoclar Vivadent, Schaan, Liechtenstein).	High-viscosity bulk-fill resin composite	Bisphenol-A-glycidylmethacrylat (Bis-GMA), Bis-EMA, and barium glass filler
Group 3 Viscalor bulk	VOCO, Cuxhaven, Germany	The composite was preheated using its specialized heating gun and injected directly from the compule into the cavity using bulk-fill technique.	Thermoviscous bulk-fill resin composite	Bis-GMA, aliphatic dimethacrylate, and inorganic filler
Group 4 Cention Forte	Ivoclar Vivadent, Schaan, Liechtenstein	The material was prepared in a capsule mixer for 15 s and applied directly from the capsule with a capsule gun using the bulk-fill technique. Then, it was shaped with an Optrasculpt modeling instrument.	Moderate-viscosity alkasite material	Calcium-fluoro-silicate glass, barium-aluminosilicate glass, ytterbium trifluoride, copper salt and thiocarbamide-self cure initiator (Ivocerin), acyl phosphine oxidephotoinitiator, pigment, urethane dimethacrylate (UDMA), tetramethyl xylylendiurethane dimethacrylate, tricyclodecandimethanol dimethacrylate (DCP), polyethylene glycol 400 dimethacrylate (PEG400 DMA), initiator (hydroperoxide—self-curing), and stabilizer
Group 5 Dyract XP (control)	Dentsply Sirona, Konstanz, Germany	The material was applied from the compule in two layers of 2 mm, condensed and shaped with Optrasculpt modeling instrument.	High-viscosity compomer material	UDMA, carboxylic acid modified dimethacrylate, TEGDMA, trimethacrylate resin (TMPTMA), dimethacrylate resins, camphorquinone, ethyl-4 (dimethylamino) benzoate, butylated hydroxy toluene (BHT), strontium-alumino-sodium-fluoro phosphor-silicate glass, highly dispersed silicon dioxide, strontium fluoride, iron oxide pigments, and titanium oxide pigments

## Data Availability

The original contributions presented in this study are included in this article, and further inquiries can be directed to the corresponding author.
